# Supplementary material for: Angelica sinensis polysaccharide as potential protectants against recurrent spontaneous abortion: focus on autophagy regulation
Source: Front Med (Lausanne). 2025 Jan 15;12:1522503. doi: 10.3389/fmed.2025.1522503 (PMC11774876; doi:10.3389/fmed.2025.1522503)
Supplement: Supplementary material S1 — The main instruments used during the LC-MS process, along with their models/specifications and manufacturers. [file Supplementary_file_1.zip › Metabolomics sequencing data FC1.2/8.代谢通路分析/Exposure_Ctrl/all/KEGG/mmu00010.html]

KEGG PATHWAY: Glycolysis / Gluconeogenesis - Mus musculus (house mouse)


# Glycolysis / Gluconeogenesis - Mus musculus (house mouse)


[
Pathway menu
| Organism menu
| Pathway entry
| Show description
| Download
| Help
]

Glycolysis is the process of converting glucose into pyruvate and generating small amounts of ATP (energy) and NADH (reducing power). It is a central pathway that produces important precursor metabolites: six-carbon compounds of glucose-6P and fructose-6P and three-carbon compounds of glycerone-P, glyceraldehyde-3P, glycerate-3P, phosphoenolpyruvate, and pyruvate [MD:M00001]. Acetyl-CoA, another important precursor metabolite, is produced by oxidative decarboxylation of pyruvate [MD:M00307]. When the enzyme genes of this pathway are examined in completely sequenced genomes, the reaction steps of three-carbon compounds from glycerone-P to pyruvate form a conserved core module [MD:M00002], which is found in almost all organisms and which sometimes contains operon structures in bacterial genomes. Gluconeogenesis is a synthesis pathway of glucose from noncarbohydrate precursors. It is essentially a reversal of glycolysis with minor variations of alternative paths [MD:M00003].


##### Option

Scale:


100%

Image resolution:


 High

##### Search

##### ID search

##### Color

##### Module

Complete only
Including 1 block missing
Including any incomplete

- Pathway modules
  - Carbohydrate metabolism
    - Central carbohydrate metabolism
      - M00001
        Glycolysis (Embden-Meyerhof pathway)
      - M00002
        Glycolysis, core module involving three-carbon compounds
      - M00003
        Gluconeogenesis
      - M00307
        Pyruvate oxidation


KGML

Image (png) file 1x
